# Geochemical Evidence of the Seasonality, Affinity and Pigmenation of *Solenopora jurassica*

**DOI:** 10.1371/journal.pone.0138305

**Published:** 2015-09-14

**Authors:** Holly E. Barden, Julia Behnsen, Uwe Bergmann, Melanie J. Leng, Phillip L. Manning, Philip J. Withers, Roy A. Wogelius, Bart E. van Dongen

**Affiliations:** 1 Williamson Research Centre for Molecular Environmental Science, School of Earth Atmospheric and Environmental Sciences, University of Manchester, M13 9PL, Manchester, United Kingdom; 2 School of Materials Science, University of Manchester, M13 9PL, Manchester, United Kingdom; 3 SLAC National Accelerator Laboratory, Menlo Park, CA 94025, United States of America; 4 NERC Isotope Geosciences Facilities, British Geological Survey, Keyworth, Nottingham NG12 5GG, United Kingdom; 5 Centre for Environmental Geochemistry, School of Geography, University of Nottingham, Nottingham, NG7 2RD, United Kingdom; University of California Davis, UNITED STATES

## Abstract

*Solenopora jurassica* is a fossil calcareous alga that functioned as an important reef-building organism during the Palaeozoic. It is of significant palaeobiological interest due to its distinctive but poorly understood pink and white banding. Though widely accepted as an alga there is still debate over its taxonomic affinity, with recent work arguing that it should be reclassified as a chaetetid sponge. The banding is thought to be seasonal, but there is no conclusive evidence for this. Other recent work has, however demonstrated the presence of a unique organic boron-containing pink/red pigment in the pink bands of *S. jurassica*. We present new geochemical evidence concerning the seasonality and pigmentation of *S. jurassica*. Seasonal growth cycles are demonstrated by X-ray radiography, which shows differences in calcite density, and by varying δ^13^C composition of the bands. Temperature variation in the bands is difficult to constrain accurately due to conflicting patterns arising from Mg/Ca molar ratios and δ^18^O data. Fluctuating chlorine levels indicate increased salinity in the white bands, when combined with the isotope data this suggests more suggestive of marine conditions during formation of the white band and a greater freshwater component (lower chlorinity) during pink band precipitation (δ^18^O). Increased photosynthesis is inferred within the pink bands in comparison to the white, based on δ^13^C. Pyrolysis Gas Chromatography Mass Spectrometry (Py-GCMS) and Fourier Transform Infrared Spectroscopy (FTIR) show the presence of tetramethyl pyrrole, protein moieties and carboxylic acid groups, suggestive of the presence of the red algal pigment phycoerythrin. This is consistent with the pink colour of *S. jurassica*. As phycoerythrin is only known to occur in algae and cyanobacteria, and no biomarker evidence of bacteria or sponges was detected we conclude *S. jurassica* is most likely an alga. Pigment analysis may be a reliable classification method for fossil algae.

## Introduction

*Solenopora jurassica*, often referred to as the ‘beetroot stone’ is particularly striking due to its pink and white banding. The organism is thought to have inhabited warm shallow water environments, with exposure to occasional storms [1]. Geologically important as a reef-building organism, alongside corals, it is also biologically important as the mechanism of its banding pattern is still poorly understood. It remains unclear whether it is due to seasonal changes in the environment or caused by other factors. Various mechanisms have been suggested, most proposing that they represent seasonal growth bands [1, 2]. Thus far, however, there is no conclusive evidence of this. Originally classified by Dybowski in 1877 as a chaetetid sponge, it has since been accepted as a fossil calcareous alga [1, 3], though this taxonomic assignment has been questioned [4]. Cellular structure is clearly identifiable; elongated cells radiate from the inner core of the thallus to its exterior in the direction of growth [1]. The visible bands have been shown to correlate with the degree of cellular preservation [1–3]. Pink bands are composed of poorly preserved cells, with detail obliterated by extensive recrystallization [1], whereas white bands are composed of relatively better preserved cells. The mechanism of this preservational difference and its relationship to the visible banding pattern remains unclear.

Harland and Torrens [1] proposed that the pigment had been leached out of the white bands due to the increased porosity created by better cellular preservation. The pink band was thought to be less porous due to the recrystallisation of the cellular structure creating a more densely packed material than the well ordered structure of the white band. A mechanism for this difference in cellular preservation was outlined by Wright [2], in which he argued for its dependence on magnesium levels in cell wall calcite. Extant calcareous algae have been shown to have different amounts of magnesium in their cell walls depending on growth rate [5]. During periods of slower growth the magnesium uptake by the cells for photosynthesis and respiration is low and the preferred Mg/Ca ratio in the calcite is easily maintained by diffusion. When growth is faster the diffusion of magnesium is not fast enough to replace the amounts used by the cells and so the ratio cannot be maintained. This leads to more magnesium being stored in the calcite during periods of slower growth than periods of faster growth. Wright [2] proposed that this occurred in the tissues of *S. jurassica*, and that the pink bands retained pigment because they contained higher levels of magnesium in life and therefore during diagenesis the conversion of high magnesium calcite to low magnesium calcite caused extensive degradation in cellular structure. Wright [2] predicted that the thicker pink bands in *S. jurassica* were deposited in the summer months and the white bands in the winter months, however despite electron microprobe analysis no evidence of magnesium level variation was found [2].

Until very recently nothing was known about the pigment present within the pink bands of *S. jurassica*, other than that it had been shown to be organic [6] and suggested to be a porphyrin, preserved due to the rapid burial of the algae by sediments [1]. Wolkenstein et al. [7] have recently isolated a novel boron containing organic pigment, the ‘borolithochrome’, from *S. jurassica* fossils. When concentrated it exhibits a bright red/pink colour and is composed of a highly condensed aromatic system with the boron bound to phenolic moiety ligands; it has no known modern analogue.

The aims of this work are broadly to attempt to use biomarker analysis to study the pigments in *S. jurassica*, to determine its affinity and detemine why it displays a banded pattern. In particular we aim to analyse the chemistry of the fossil, and establish whether any pigments are present that might be diagnostic of algae, such as the red algal pigment-protein complex phycoerythrin. This would establish the classification of fossil algae by their pigments, as is done for modern algae [8]. If successful this technique could be applied more widely in the fossil record. We also aim to determine whether the previous mechanism for banding by magnesium levels in cell wall calcite is valid and whether there is any evidence of seasonality. Furtherore, establishing that the fossil was subject to seasonal changes in temperature would help with palaeoenvironmental interpretations.

## Materials and Methods

### Samples and preparation

**Fig 1:**
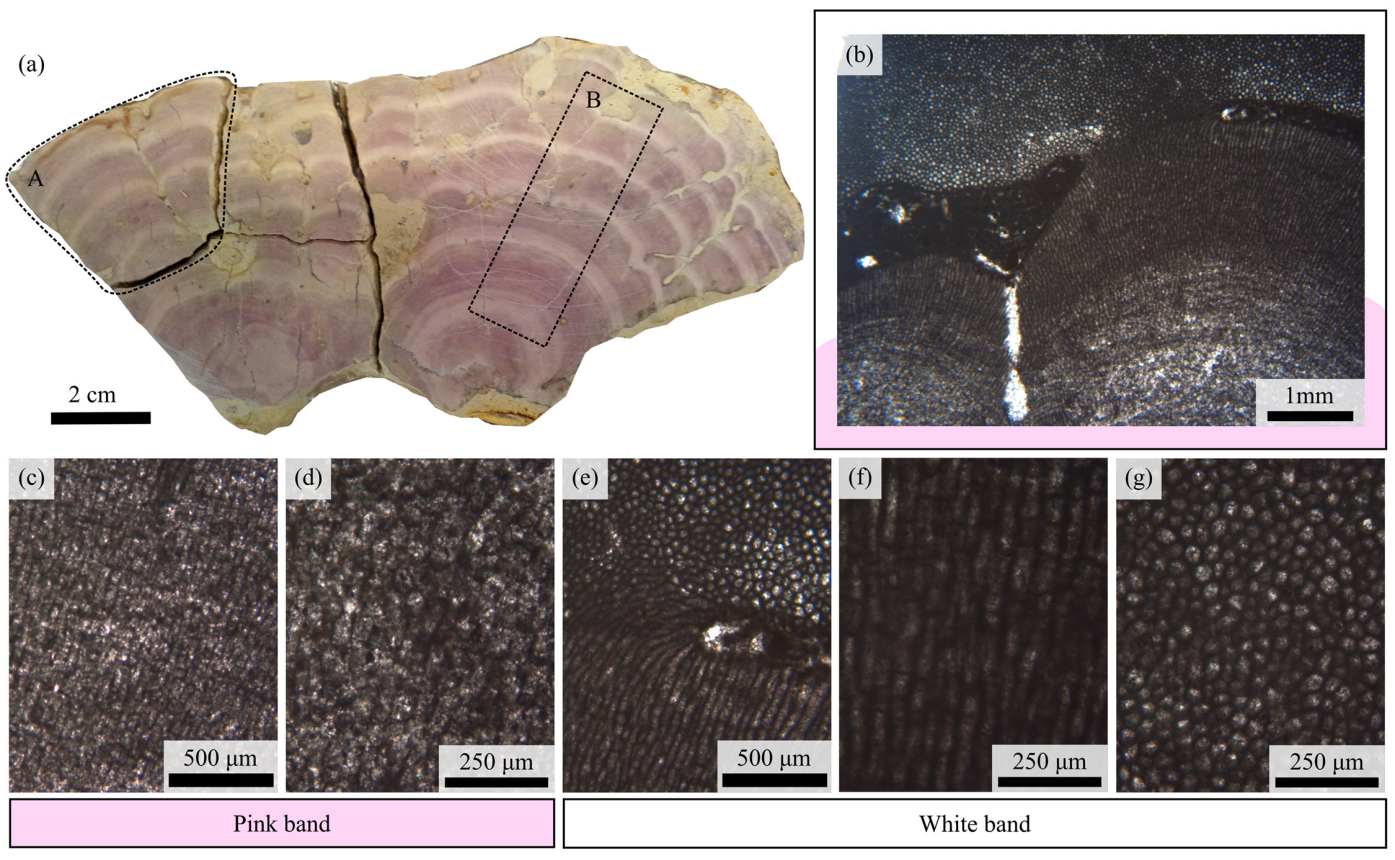
Photographs of *Solenopora jurassica* (UOM-232) used in the study. (a) Thick section of UOM-232. A, denotes the section subsequently analysed by 2D X-ray radiography, and B, the area mapped by XRF. (b-g) Light microscopy images of a thin section of UOM-232 showing the pink (b-c) and white (e-f) bands at different magnifications. The difference in cellular preservation between the two bands can be clearly seen under the light microscope, with the cellular structure clearly discernible in the white bands but not the pink ones.

The sample used in this study (sample number UOM-232; Fig 1) is from the collections of the University of Manchester. It was collected from the White Limestone Formation (Middle and Upper Bathonian) of Foss Cross quarry near Chedworth in Gloucestershire, a site famous for such specimens. The sample was collected over 20 years ago and no specific collections details or permit information for the sample exists. The depositional environment has been described as moderate to high energy shoaling with occasional storms [1]. Based on the work of previous authors on similar fossils [1–3] we can confidently assign this specimen to *S. jurassica*. The thick section cut for analysis by infrared and chromatography techniques was taken from the interior of the specimen and the outer edges were sanded down. The drill tip and then the whole section were rinsed in dichloromethane to minimize the chances of contamination from handling of the sample during collection and display. This section was then ground to a powder for analysis. Subsequently the main block and all sections taken were kept in foil packets and sealed in plastic bags to avoid further contamination. Standard petrographic thin sections were prepared and studied under a Nikon Optiphot confocal microscope.

### Extraction and decalcification

100 g of powdered bulk sample was extracted with dichloromethane:methanol (DCM:MeOH, 2:1, v/v) for 24 h using Soxhlet equipment. Colourless total lipid extracts (TLE) were recovered and concentrated by using rotary evaporation. Bulk and residue powder samples were reacted with 10% HCl to remove calcium carbonate and thereby concentrate organic material. After no further reaction was observed with the acid the samples was washed in deionized water, centrifuged, excess water pipetted off, and then freeze dried to remove all remaining water. Approximately 99% of the mass was lost in the process [9]. Bulk and residue samples were then analysed by Py-GCMS. Samples were white in appearance before and after extraction, but went brown after decalcification.

### Electron Microprobe (EPMA)

A section was cut and half of it etched with 1% HCl for 5 min then thoroughly rinsed with deionized water and air dried. It was then glued into the bottom of a circular aluminium mount and ground so that the surface was flush with the top. The sample was carbon coated and silver dag applied to one edge to draw current from the surface. A Cameca SX 100 microprobe in Wavelength Dispersive Spectrometry (WDS) mode was used to detect and map elements on the surface of the sample. K, S, Ca and Cl were analysed using a pentaerythritol (PET) crystal spectrometer, P, Si and Mg using a thalium acid pthalate (TAP) crystal spectrometer, and Zn, Cu, Fe and Mn using a lithium fluoride detector (LIF) crystal spectrometer. All elements were quantified using their respective Kα characteristic emission line. 400 μm^2^ maps of each element were taken of 0.5 μm^2^ resolution, as well as 30 point analyses in the pink bands, 30 on the cell walls and 30 on the cell vacuoles of the white band to determine abundance. The electron beam accelerating voltage was 15 kV with a current of 20 nA: excitation volume was approximately 1 μm^3^.

### X-ray Photoelectron Spectroscopy (XPS)

The XPS spectra were recorded using a Kratos Axis Ultra spectrometer employing a monochromated Al Kα X-ray source and an analyser pass energy of 80 eV for survey scans and 20 eV for elemental scans, resulting in a total energy resolution of ca. 1.2 eV—1.4 eV or 0.6 eV—0.7 eV respectively. Uniform charge neutralisation of the photoemitting surface was achieved by exposing the surface to low energy electrons in a magnetic immersion lens system (Kratos Ltd.). The system base pressure was 1 × 10^-9^ mBar. Spectra were analysed by first subtracting a Shirley background and then obtaining accurate peak positions by fitting peaks using a mixed Gaussian/Lorenzian (30/70) line shape. Quantification of surface atom% was achieved using a derived analyser transmission function and Scofield theoretical elemental cross-sections. During fitting, spin orbit split components were constrained to have identical line width, elemental spin orbit energy separations and theoretical spin orbital area ratios. All photoelectron binding energies (BE) are referenced to C1s peaks set at 285 eV BE. The analyser was calibrated using elemental references; Au 4f7/2 (83.98 eV BE), Ag3d5/2 (368.26 eV BE) and Cu2p3/2 (932.67 eV BE).

### Isotope analysis

Twenty-five samples were taken across the sample with between 3 and 7 sample holes per band taken with a small hand drill with a 1mm drill bit. Approximately 30-100 microgrammes of carbonate was used for isotope analysis using a IsoPrime dual inlet mass spectrometer plus Multiprep device. Isotope values (δ^13^C, δ^18^O) are reported as per mil (‰) deviations of the isotopic ratios (^13^C/^12^C, ^18^O/^16^O) calculated to the VPDB scale using a within-run laboratory standard calibrated against NBS-19 standards. Analytical reproducibility of the standard calcite (KCM) is <0.1 ‰ for δ^13^C and δ^18^O.

### Pyrolysis Gas Chromatography Mass Spectrometry (Py-GCMS)

2 mg of the sample was analysed using normal flash Py-GCMS. Samples were pyrolysed with a CDS (Chemical Data Systems) 5200 series pyroprobe pyrolysis unit by heating at 600°C for 20 s. The fragmented macromolecular components were analysed using an Agilent 7890A gas chromatograph fitted with a HP fused column (J+W Scientific; 5% diphenyl-dimethylpolyolsiloxane; 30 m, 0.32 mm i.d., 0.25 μm film thickness) coupled to an Agilent 5975C MSD single quadrupole Mass Spectrometer operated in electron ionization (EI) mode (scanning a range of *m/z* 45-650 at 1 scan s^-1^ with a 4 min solvent delay; ionization energy 70 eV). The pyrolysis transfer line and the injector port temperatures were set at 350°C, the heated interface at 280°C, the EI source at 230°C and the MS quadrupole at 150°C. The carrier gas was helium and the sample was introduced in split mode at a 2:1 ratio. The oven was programmed from 40°C (held for 4 min) to 300°C at 4°C min^-1^ and held at this temperature for 5 min.

Thermochemolysis of samples was carried out by adding 10 μl of tetramethylammonium hydroxide solution to a second sample, leaving it for 5 min, and then analysing it using the same equipment. The scan range was *m/z* 60-650 with a 15 min solvent delay. The oven was programmed from 40°C (held for 4 min) to 320°C at 4°C min^-1^ and held at this temperature for 5 min. Compounds were identified using the NIST database and by comparison with spectra from the literature.

### Fourier Transform Infrared Spectroscopy (FTIR)

Samples were analysed using a Perkin Elmer Spotlight 400 system (wavenumber range 4000 cm^-1^ to 900 cm^-1^). Spectra were taken with a 20 μm^2^ aperture and 4 cm^-1^ resolution; final spectra were an average of 16 scans. All spectra and maps were background subtracted. Organic peak assignments were made using the Bio-Rad KnowItAll Informatics system 8.2 Multi-Technique database, inorganic peaks by reference values [10].

### Synchrotron Rapid Scanning X-ray Fluorescence (SRS-XRF)

SRS-XRF was carried out at wiggler beamline 6-2 of the Stanford Synchrotron Radiation Lightsource (SSRL). Scans were undertaken in ambient conditions with beam energies of 13.5 keV (hard X-rays, flux approximately 8.95 × 10^10^ photons s^-1^) and 3.15 keV (soft X-rays, flux approximately 8.7 × 10^8^ photons s^-1^). A 100 μm diameter pinhole aperture was used to control beam spot size. Samples were mounted on an x-y motorised stage and signals were detected using a single element Li drifted (Vortex) Si detector. For hard X-rays the stage was at a fixed 45° incident angle to the beam and the detector was fixed at a 90° scattering angle to the beam with a 70 mm air path from the sample. The sample was 6.2 cm from the pinhole. For soft X-ray scans the samples were mounted inside a metal case, the top sealed in polythene and the case purged with flowing helium, creating a lower density atmosphere to reduce absorption of the incident and fluoresced X-rays. The stage was at a fixed 45° incident angle to the beam and the detector was fixed at a 64° scattering angle to the beam with a 15 mm air path from the sample. Point analyses were taken by locating and driving the mounted sample to a point of interest and collecting a full energy dispersive spectrum for 100 seconds. Spectra were calibrated by comparison to a point analysis for a Durango apatite standard of known elemental composition. Detection limits are approximately 1 ppm for higher atomic weight elements such as arsenic, and several weight percent for lighter elements. Errors are approximately ±4% absolute for the transition metals and ±10% absolute at lower energy. Spectra were analysed using PyMCA software version 4.4.1 [11].

### 2D X-ray radiography

A subsection of UOM-232 was taken for 2D X-ray radiographic analysis (A in Fig 1a). The radiographic images were taken on the Nikon metrology 225/320 kV custom bay system of the Henry Moseley X-ray Imaging Facility (HMXIF) with the 225 kV source and a molybdenum target using a voltage of 100 kV and a current of 100 μA. The X-ray beam was not filtered. A PerkinElmer 2000 × 2000 pixels 16-bit amorphous silicon flat panel detector was used to obtain the images. The acquisition software was Nikon Metrology proprietary software InspectX version XT 2.2 service pack 5.5.

### X-ray Diffraction (XRD)

Glancing incidence scans of the polished surface were run on a Bruker D8 Advance diffractometer using a fixed incident angle of 2.5°, with a detector step size of 0.02° at a speed of 2 s step^-1^. A Goebel mirror attachment provided parallel X-ray beam optics, and a scintillation detector with a Soller slit assembly was used. Incident beam wavelength was 1.5418 Å(Cu Kα). Peak assignment was achieved using Eva 14.0 software to compare the measured data with standards from the ICDD Powder Diffraction File.

## Results

### Light microscopy

As noted in previous studies [1, 2], the striking pink and white banding displayed on the sample (Fig 1a) correlates with the degree of cellular preservation as visible in a thin section of the sample.

The white bands are composed of relatively better preserved cells (Fig 1e–1g) than the pink bands (Fig 1b–1d), which have been reported to have lost their definition by means of extensive recrystallisation [2, 3]. A thin section (taken in the same plane as Fig 1a), shows the cells in the white bands to be for the most part to be longitudinal, the long axis indicating the direction of growth (Fig 1f), usually radially outwards from the centre [1]; however horizontal (Fig 1g) and vertical growth can be seen occurring in the same cross section (Fig 1b and 1e). This may indicate a response to a sediment infill or covering of the thallus (Fig 1b and 1e). Average length of cells is 264 μm and diameter is 64 μm (+/- 18 nm, average of 10 measurements). The average width of the white band is also significantly smaller (3.83 mm) than the pink band (9.38 mm) (+/- 18 nm, average of 45 measurements, p = <0.0001, Mann Whitney U test).

### EPMA

**Fig 2:**
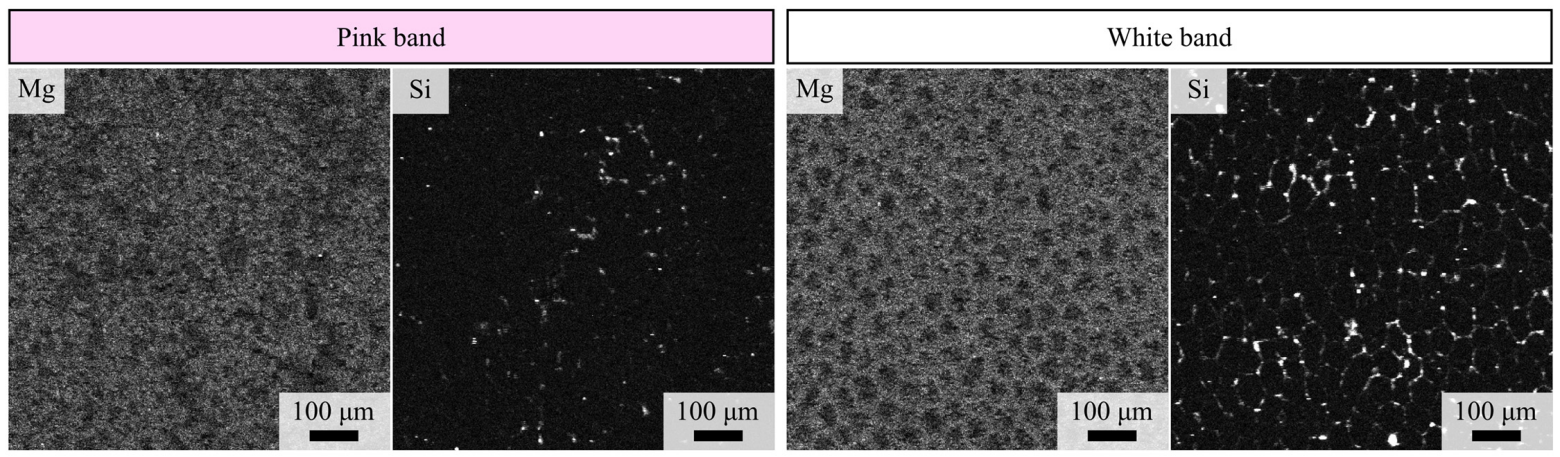
Electron Microprobe images of UOM-232. The Mg and Si is concentrated in the cell walls and highlights the much better cellular preservation in the white bands compared to the pink bands. White represents high abundances and black represents low abundances.

X-ray fluorescence maps (Fig 2 and S1 Fig) and point analyses (Table 1) using EPMA indicates that Si, S, Mg and Fe are preferentially bound within the cell walls of the white band. Patterns of abundance for other elements are less clear from mapping, but point analyses indicate that levels of, Si, S and Mg are significantly higher in the cell walls of the white band than in the pink band (p<0.001 Mann Whitney U test), but levels of Mg and Fe are significantly lower in the white band cell vacuole than in the pink band (p<0.01 Mann Whitney U test). The cellular preservation in the pink band was too poor to enable differentiation of cell wall and vacuole. The Mg/Ca molar ratio was higher in the white band cell wall (12.39 mmol mol^-1^) than the pink band (7.86 mmol mol^-1^), potentially indicating a higher temperature during the time the white band was deposited [12].

**Table 1:** Elemental analysis of UOM-232 by electron microprobe analysis (EPMA).

Element	White band vacuole (ppm)	White band cell wall (ppm)	Pink band (ppm)	Significance between white band cell vacuole and pink band	Significance between white band cell wall and pink band
P	3290	3030	3360.00	>0.05	<0.001
Si	74.0	1860	107	>0.05	<0.001
S	834	1780	879	>0.05	<0.001
Mg	1240	2870	1950	<0.01	<0.001
Ca	409000	382000	409000	>0.05	<0.001
Mn	135	146	180	BDL	BDL
Fe	889	2460	1240	<0.01	>0.05
Cu	72.4	67.1	98.3	BDL	BDL
Zn	96.9	95.9	64.3	>0.05	>0.05
K	22.0	138	13.0	BDL	BDL
Cl	48.3	131	54.7	BDL	BDL

Elemental abundances in pink and white bands indicated by EPMA. BDL indicates a value is below the detection limit for that element.

### XPS

**Fig 3:**
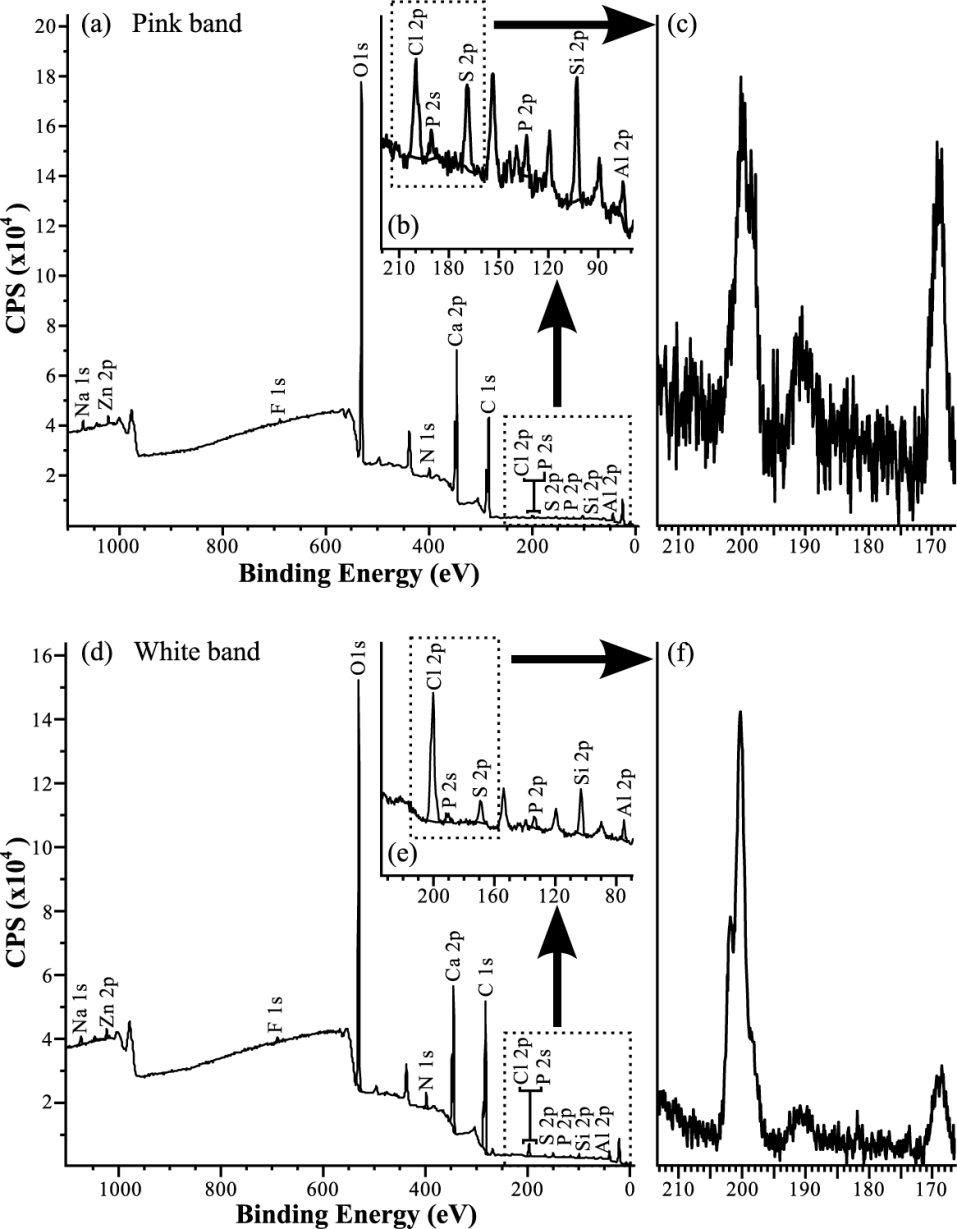
XPS spectra of the pink and white bands of UOM-232. Wide energy range scans are shown in (a) and (d), (b) and (e) show scans of 220 eV—70 eV, and (c) and (f) show high resolution scans of the Cl 2p, P 2s and S 2p peaks. Due to the presence of phosphate the phosphorus peak 2s peak occurs in the same range as the boron peak (190 eV—191 eV) meaning boron could not be resolved.

Wide scans (Fig 3a and 3d) show the presence of many different elements in both bands, the highest in abundance being Ca, C and O, due to the sample being predominantly composed of calcite (Table 2). Errors on peak amplitudes are estimated to be approximately 50%, therefore the data is treated qualitatively rather than quantitatively. In both bands fine scans of the P 2p peak at circa 130 eV (Fig 3b and 3e) shows the presence of a phosphate peak at 133.5 eV. The P 2s peak would typically occur at approximately 188 eV, however due to the fact that phosphorous is present as a phosphate complex, this induces a shift and make this peak appear at 190 eV—191 eV, exactly the same binding energy range at which we expect to find the borate B 1s peak. Higher resolution scans (Fig 3c and 3f) were no more successful at separating 2 peaks in this region. XPS analysis did not detect boron within UOM 232.

**Table 2:** Quantification data of elements present in UOM-232 shown by X-ray photoeletron spectroscopy.

Element and orbital	% conc. pink band	% conc. white band
Na 1s	0.22	0.18
Zn 2p	0.11	0.15
F 1s	0.17	0.26
O 1s	37.81	33.76
N 1s	1.41	1.85
Ca 2p	11.31	8.32
C 1s	46.10	51.32
Si 2p	1.16	1.62
Cl 2p	0.41	1.32
S 2p	0.48	0.66
Al 2p	0.60	0.34
P 2p	0.21	0.23

### Isotope analysis

**Fig 4:**
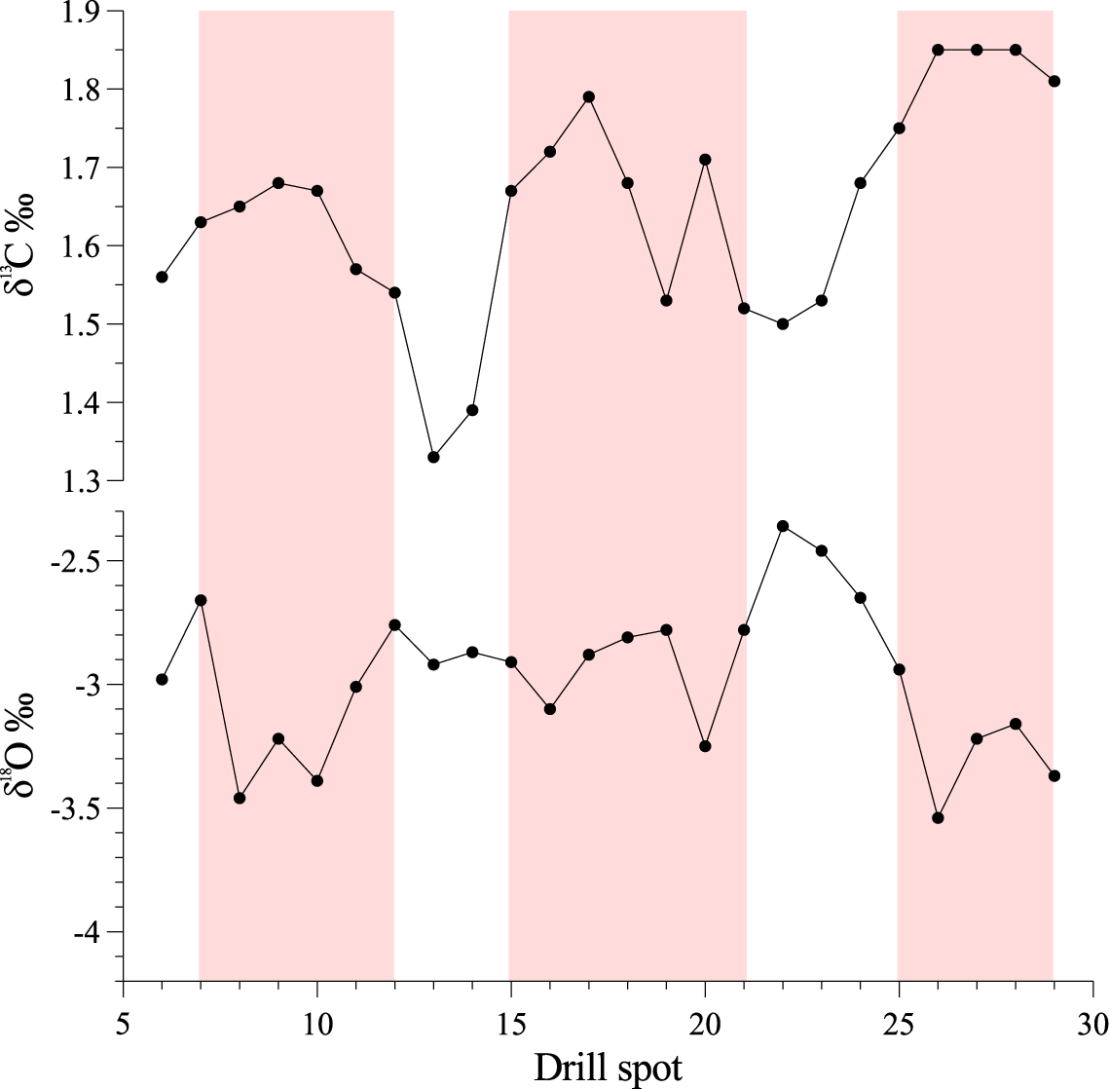
Isotope data from UOM-232 showing the white and pink (shaded) bands and the location of the drill spots.

Equilibrium δ^18^O of calcium carbonates decreases by about by about 0.24 ‰ for each 1°C increase in temperature [13]. Therefore, converting the changes we see in the δ^18^O values of UOM-232 to temperatures indicates that the white bands were deposited during temperatures approximately 4°C cooler than those when the pink layers precipitated. The δ^18^O data does not, however, indicate as clear a pattern of cyclicity as the δ^13^C levels (Fig 4), which increases in the pink bands and decreases in the white bands. The difference in cyclicity patterns may be a function of different preservation, which tends to impact on δ^18^O more readily than δ^13^C.

### Py-GCMS

**Fig 5:**
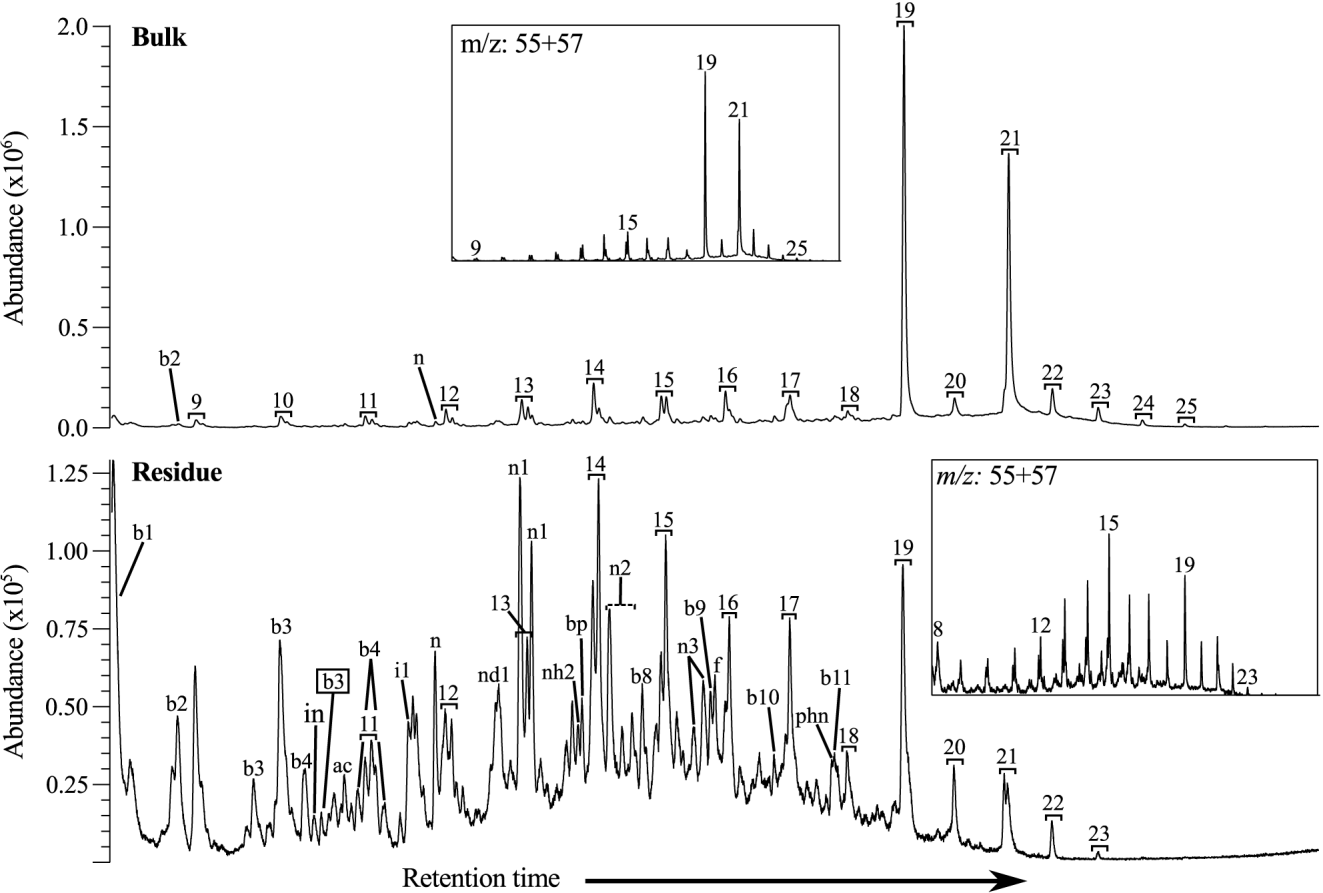
Py-GCMS total ion current and *m/z* 55+57 chromatograms of the bulk and Residue of UOM-232. The insets are not to scale. Black brackets represent *n-*alkane/*n-*alkene doublets (numbers indicate carbon chain length), ac acetophenone, bp biphenyl, f fluorene, in indane, phn phenathrene/anthracene; and bx benzene and nx napthalene derivatives, where x represents the number of carbon atoms in the alkyl group. A black outline indicates the presence of a double bond.

The bulk sample Py-GCMS Total Ion Current (TIC) chromatogram is dominated by an *n-*alkane/*n-*alkene doublet pattern ranging from C_9_ to C_25_ with the maxima at C_19_ (Fig 5). The *n-*alkane/*n-*alkene doublet pattern in the residue has a substantially different distribution pattern, dominated by lower carbon chain length moieties, C_8_ to C_23_, with the maximum at C_15_. These differences are likely caused by the extraction, indicating that these moieties are not part of an aliphatic macromolecular complex, such as those observed in fossilised plant material [14, 15]. The other products detected in the residue are mostly benzene and napthalene derivatives.

**Fig 6:**
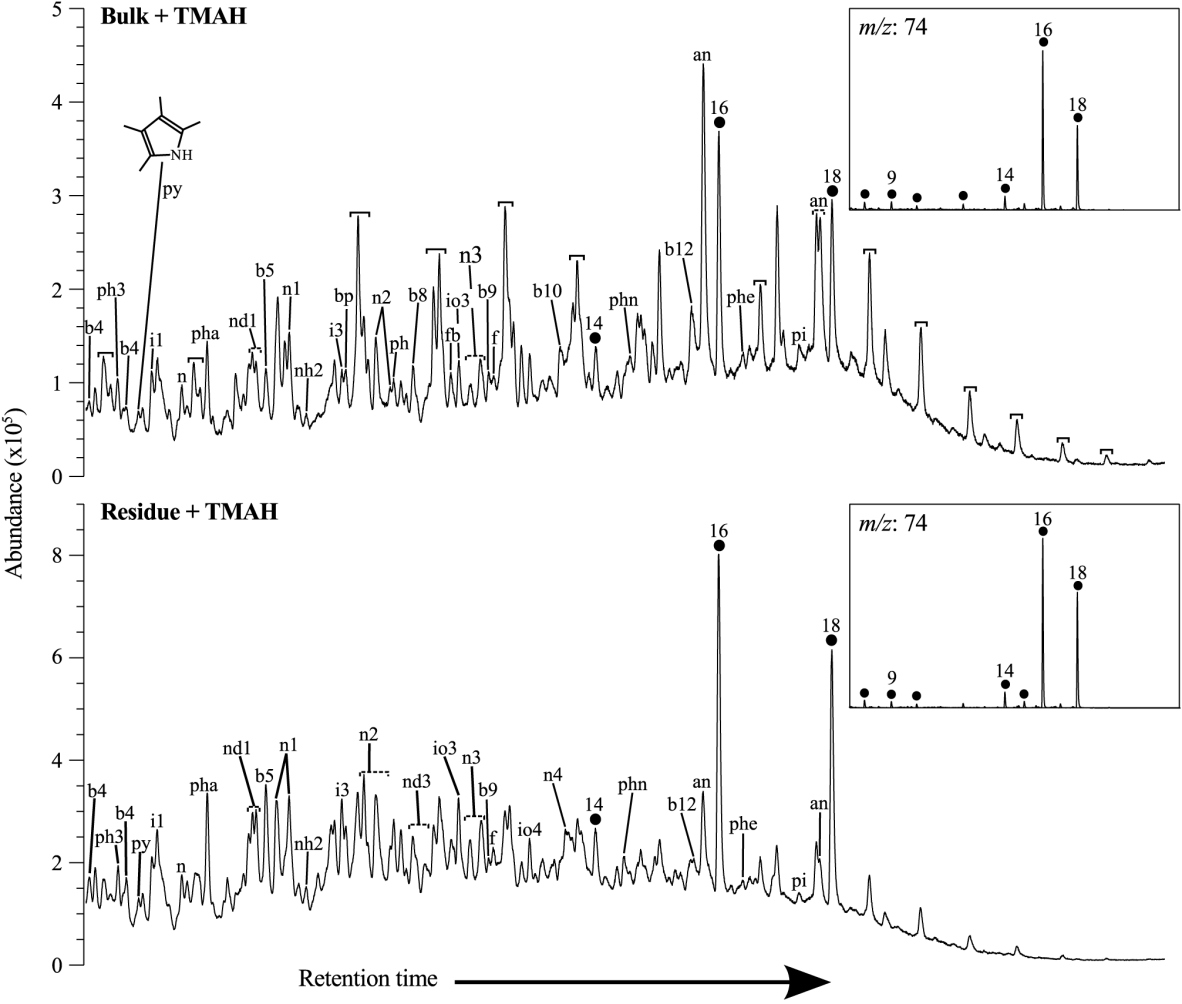
Py-GCMS Total ion current chromatograms of the bulk and residue of UOM-232 after thermochemolysis, insets (not to scale) are the *m/z* 74 mass chromatograms. Fatty acid moieties (measured as methyl esters) are represented by filled circles and *n-*alkane/*n-*alkene doublets by black brackets, numbers indicate carbon chain length; an alkane nitrile, bp biphenyl, f fluorene, fb trifluoromethylbenzoic acid pentadecylester, pha phenol-(dimethylamino), phe phenylalanine 4-amino-N-t-butyloxycarbonyl-t-butylester, phn phenthrene, pi phenol 4 4’-(1-methylethylindene)bis(2-methyl), py pyrrole, 2,3,4,5-tetramethyl; and bx benzene, ix indene, iox indole, nx napthalene, ndx dihydronapthalene, nhx tetrahydronapthalene and phx phenol derivatives, where x represents the number of carbon atoms in the alkyl group.

Py-GCMS following TMAH enhanced thermochemolysis shows the main difference after extraction of the bulk material to be a lack of *n*-alkane/*n*-alkenes, and the reduction of alkane nitriles (Fig 6), in line with the patterns observed in normal pyrolysis. The signals detected in the bulk and residue are otherwise very similar, the only other significant difference being the lack of biphenyl and trifluoromethylbenzoic acid, pentadecylester in the residue. This indicates that the majority of the material in UOM-232 is kerogenous. The presence of protein is suggested by the detection of phenol-(dimethylamino) and the amino acid phenylalanine. The fatty acid distribution is unchanged after extraction and ranges from C_8_ to C_18_, with maxima at C_16_ and C_18_ (Fig 6 insets). This pattern is very different to that of, for instance, fossil leaves [15], supporting the absence of higher plant derived material. The fact that the TLE was colourless and the residue was brown after decarbonation, indicates that the pigment responsible for the pink colour bands is part of the kerogen fraction. No bacterial hopane groups were detected.

### FTIR

**Fig 7:**
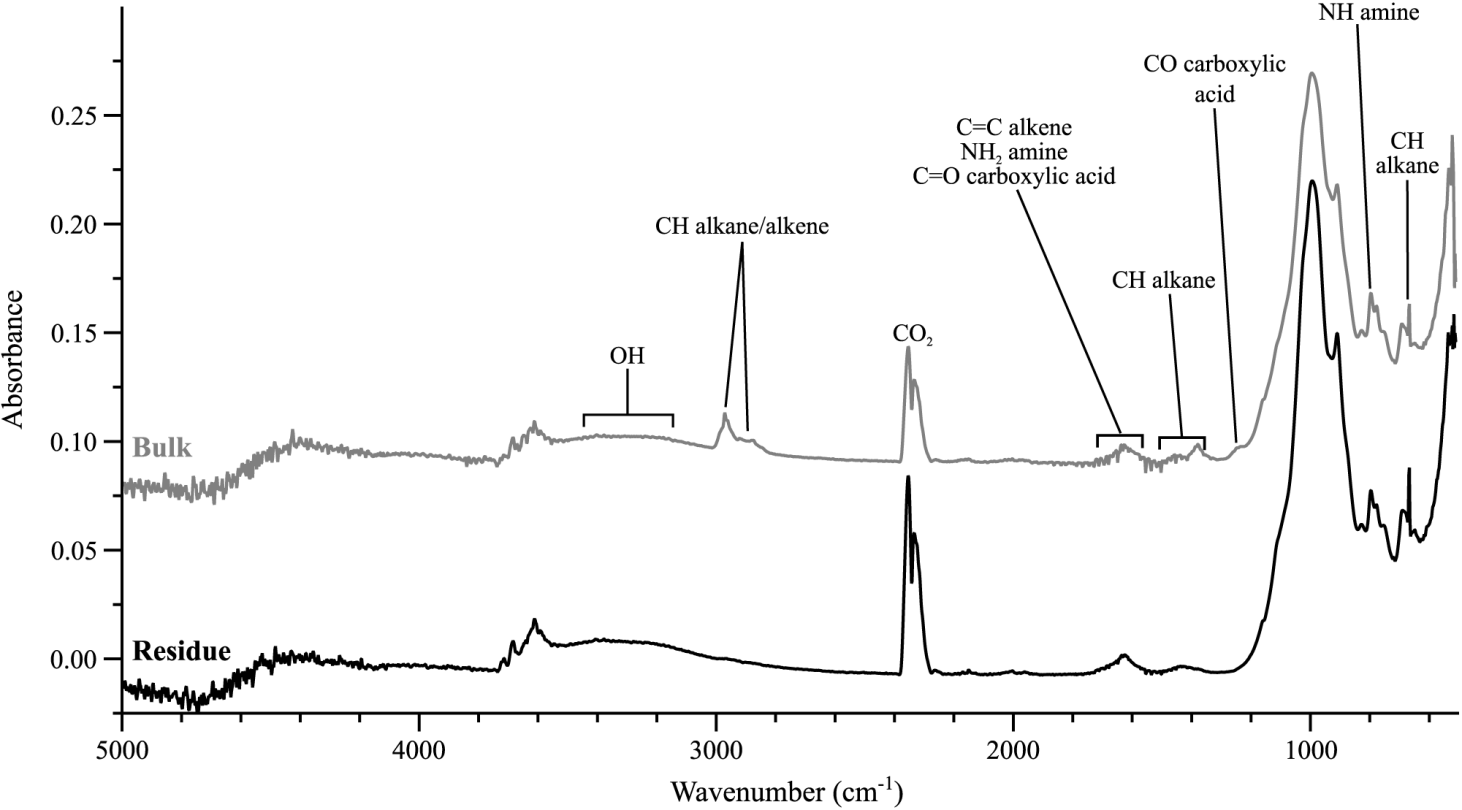
FTIR spectra of the bulk and residue of UOM-232. The major peak at approx. 1000 cm^-1^ is inorganic silica. Major components of both spectra include oxygen OH, alkene CH and C = C and NH and NH_2_ amine groups. Signal at approx. 2300 cm^-1^ is atmospheric CO_2_. The major difference between the two is the lack of CH alkane/alkene groups in the residue, consistent with their removal during extraction.

There are few notable differences between the bulk material and the residue after extraction, only the removal of alkane CH groups between 2900 cm^-1^ and 3050 cm^-1^ and the apparent disappearance of the convolved alkane CH and carboxylic acid (CO) peak (Fig 7). The presence of a broad convolved peak at approximately 1300 cm^-1^ makes the identification of individual peaks in that region difficult; however in combination with others there is good evidence in both spectra of carboxylic acid, alcohol and alkene groups. There is also evidence of proteins, due to the presence of amine NH and NH_2_ groups. The large peak at 1000 cm^-1^ is inorganic silica. Full peak assignments are given in Table 3.

**Table 3:** Infrared apsorption bands of UOM-232.

Functional group	Bond	Wavenumber range (cm^-1^)	Intensity	Vibration	Bulk + HCl	Residue + HCl
Alkanes (C-(CH_3_)_3_)	CH	2972-2952	Strong	AS stretch	X	-
	CH	2882-2862	Strong	AS stretch	X	-
	CH	1475-1435	Strong	S stretch	X	?
	CH	1395-1385	Medium	AS stretch	X	?
	CH	1370-1365	Medium	Deformation	?	?
	CC	1255-1245	Medium	Deformation	?	?
	CC	1250-1200	Medium	Skel vibration	?	?
Alcohols (Ph-OH)	OH	3350-3200	Variable	Stretch	X	X
	OH	1390-1330	Medium	Deformation	?	?
	CO	1260-1180	Strong	Stretch	X	X
Amines ((R)_3_C-NH_2_)	NH	3400-3332	Medium	AS stretch	?	?
	NH	3328-3250	Medium	S stretch	?	?
	NH_2_	1650-1590	Medium—strong	Deformation	X	X
	CN	1240-1170	Weak—medium	Stretch	?	?
	CN	1038-1022	Weak	Stretch	?	?
	NH	850-750	Strong	Wagging	X	X
Alkenes (RCH = CHR cis)	CH	3040-2940	Medium	Stretch	X	X
	C = C	1662-1631	Medium	Stretch	X	X
	CH	730-650	Medium	Deformation	X	X
Carboxylic acids (COOH)	OH	3100-2900	Variable	Stretch	?	?
	C = O	1670-1650	Strong	Stretch	X	X
	OH	1440-1395	Weak	Deformation	?	?
	CO	1320-1211	Strong	Stretch	X	-
	OH	960-875	Medium	Deformation	?	?

Table of organic absorption band assignments for UOM-232. X indicates the presence of a group,—its absence, and ‘?’ that it may be present but cannot be verified. All Bio rad source data is from the Bio-Rad KnowItAll Informatics System 8.2 Multi-Technique database. AS—asymmetric, S—symmetric, Skel—skeletal

### SRS-XRF

**Fig 8:**
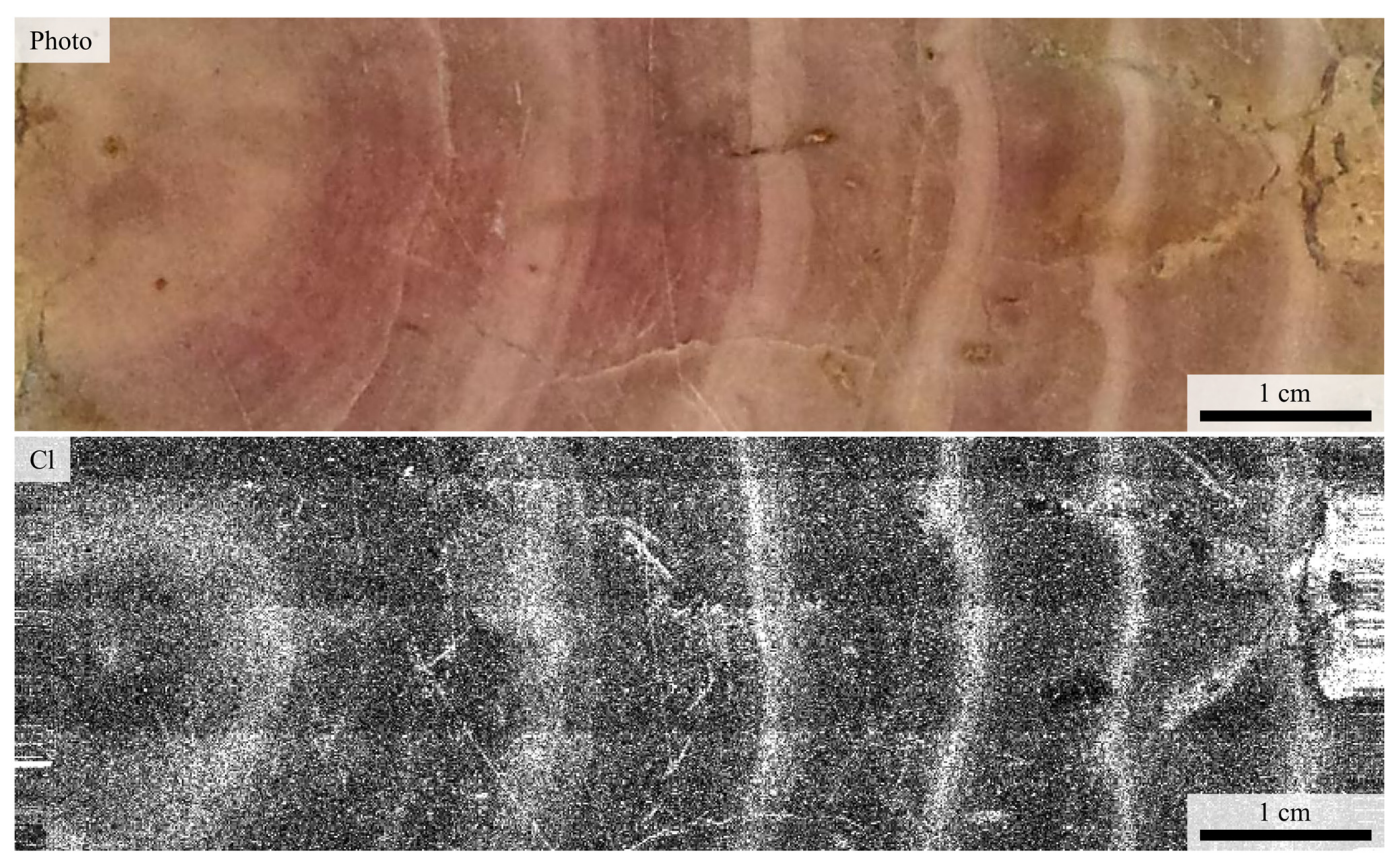
XRF map of Cl in UOM-232 with corresponding photograph in longitudinal section. Cl is clearly enriched in the white bands. White represents high abundances and black represents low abundances.

SRS-XRF mapping shows the white bands to be higher in Cl than the pink bands (Fig 8), which is supported by point analyses (Table 4). Other elements do not appear to show such strong banding although P and S also correlate with the white bands (S2 and S3 Figs). The trace metals reveal an additional zonation that is not related to the band structure.

**Table 4:** XRF point analysis of UOM-232.

Element	White band (ppm)	Pink band (ppm)
Ca	162000	163900
Ti	20.4	43.1
Mn	58.4	61.5
Fe	411	438
Ni	1.92	2.20
Cu	2.47	2.05
Zn	3.56	4.07
As	0.65	0.70
Br	0.004	0.00
Si	570	315
P	76.2	178
S	362	434
Cl	421	305

### 2D X-ray radiography

**Fig 9:**
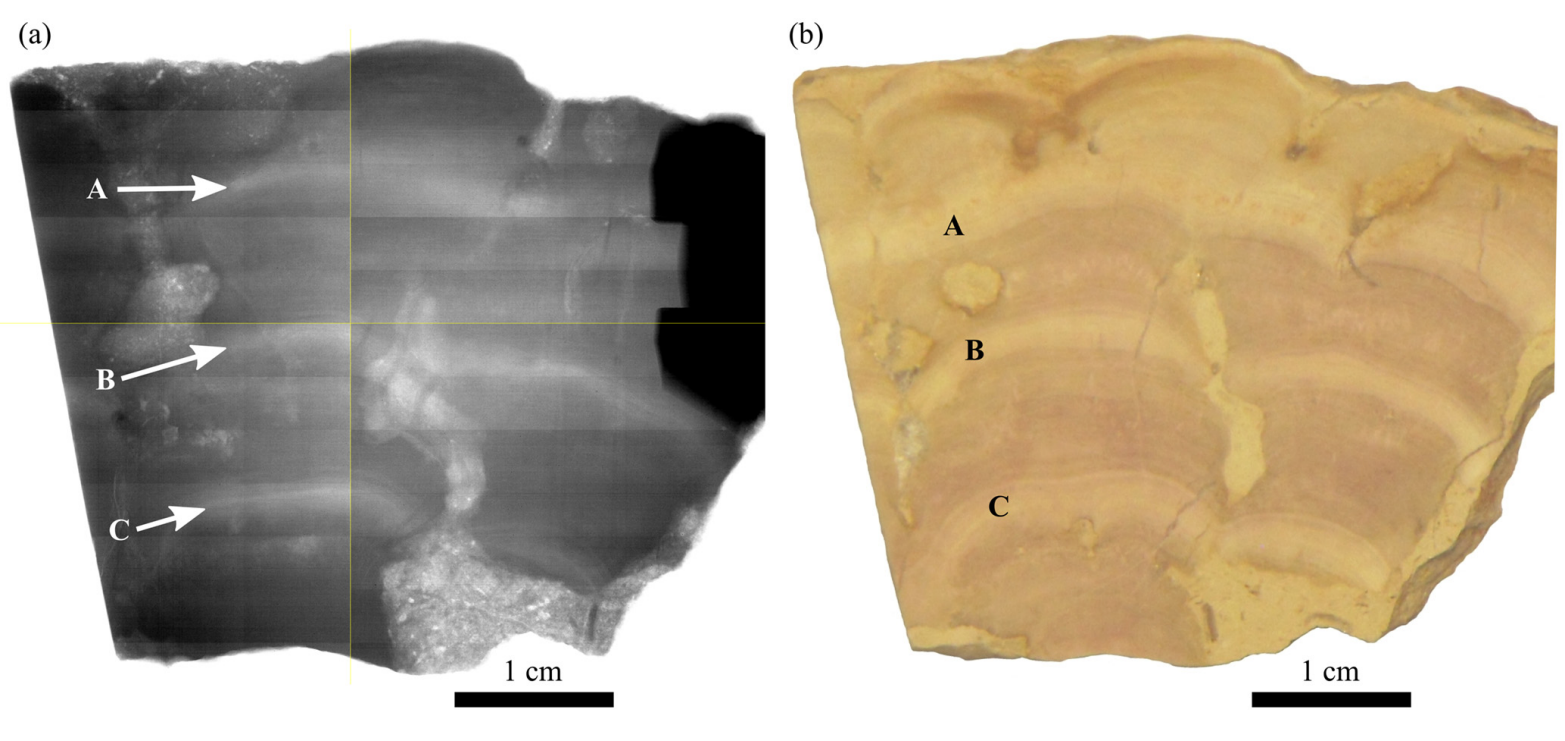
2D X-ray radiograph of UOM-232 (a) showing differences in calcite density in the different visible bands (b). Letters indicate equivalent bands on both images. The white bands are lower density than the pink bands.

2D X-ray radiographs of UOM-232 show that the white bands on the samples correspond to areas of relatively lower density calcite than the pink bands (Fig 9). This is consistent with the higher concentration of Si in the white bands since Si is not compatible in the calcite structure and will most likely be present as quartz impurities. Quartz (2.65 g cm^-3^) is lower density than CaCO_3_ (2.71 g cm^-3^. The outer layer despite being visibly white, looks to be of a similar density to the pink bands.

### XRD

**Fig 10:**
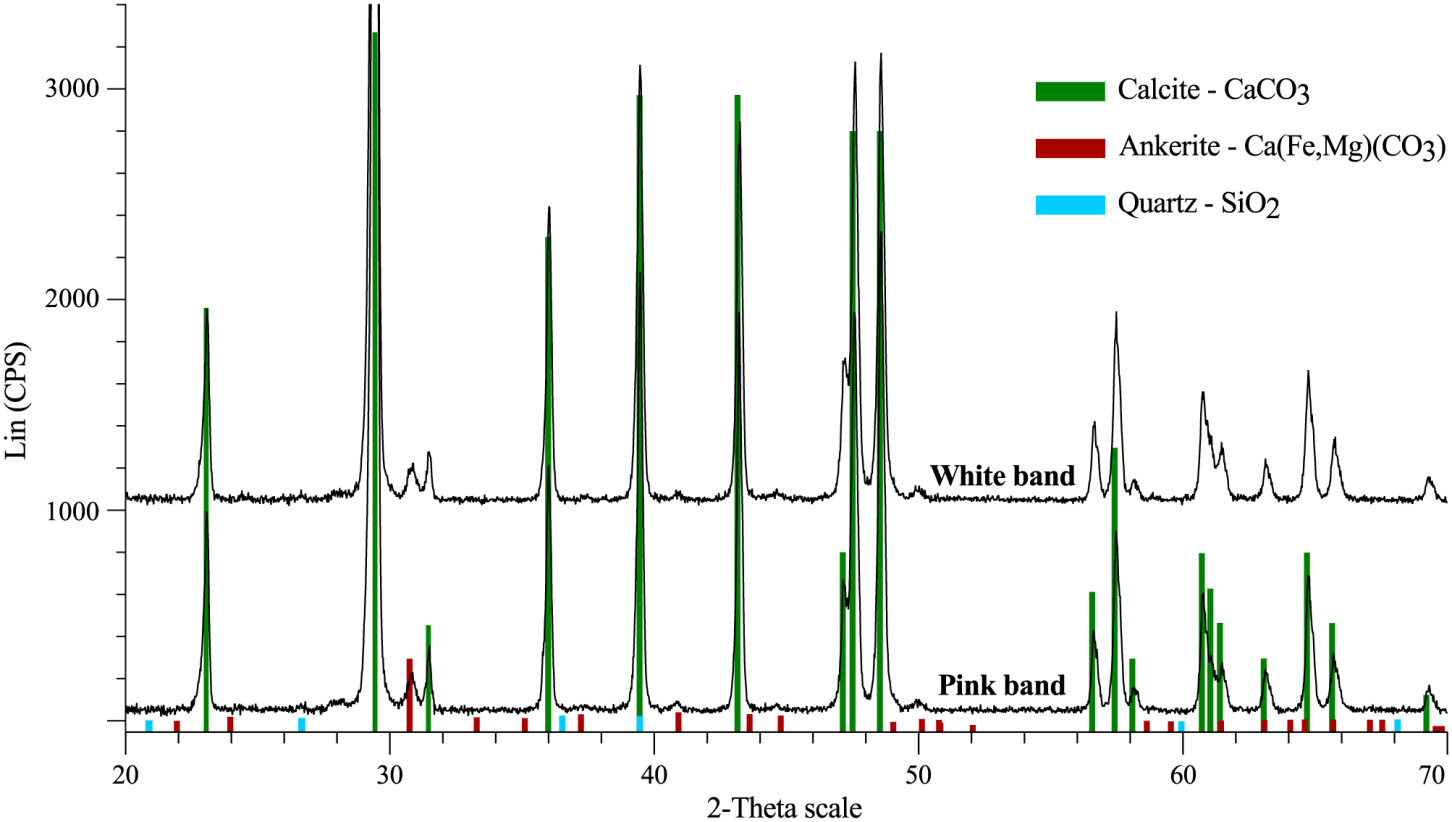
X-ray diffraction spectra of the white and pink bands of UOM-232. Both bands have the same mineralogical composition of calcite with minimal amounts of quartz and ankerite.

There appears to be no mineralogical difference between the white and pink bands. Both are composed predominantly of calcite with minimal quantities of ankerite and quartz (Fig 10).

## Discussion

### Sample curation and diagenesis

There could be some curatorial effects on the geochemical signatures of the specimen, due to it being collected some years before analysis. However, to account for this risk the work has been conducted on pristine sections of the fossil, cut so that outer surfaces that would have experienced contamination were discarded. All surfaces of the pristene sections were also washed with organic solvents to ensure the elimination of any additional organic contaminants that may have been picked up during sectioning. It should also be made clear that biomarker and pigment analysis in general looks for the breakdown products of original molecular structures, which are significantly more likely to survive than their complete precursors. The effects of diagenesis are likely to impact most strongly on the isotopic signals, particularly the δ^18^O.

### Seasonality of the banding pattern

X-ray radiography is a commonly used technique to observe seasonally determined density banding in fossil and modern corals (see Barnes and Lough [16] for a review). It was first noted by Knutson [17] that such analysis of vertical cross sections of corals showed regularly alternating bands of different calcite density, one high and one low density band representing one year’s growth. This change in density is interpreted to be the result of changing environmental conditions. However, a number of different variables are known to affect coral growth so it is difficult to narrow down the precise cause of this pattern. The phenomenon has since been noted and confirmed in over 1000 different extant species [18], and also in some fossil species [19, 20]. The 2D X-ray radiograph of sample UOM-232 clearly shows the same pattern of regularly alternating bands of low and high density (Fig 9), the pink bands being of a higher density than the white bands. Despite the fact that this technique has so far been applied to corals and not calcareous algae, the fact that both deposit a calcium carbonate framework implies that it could be applicable to both. The largest survey of density banding in extant coral found that the high density bands were associated with relatively higher water temperatures [18], but there are many examples of species that show the opposite pattern [20–22]. There is also considerable variation in the size of growth bands within individuals of the same species and well banded specimens were found where there was little seasonal variation in water temperature [18].

Temperature data/ranges calculated from both Mg/Ca and δ^18^O are also hard to interpret and give conflicting results. Higher Mg/Ca in the white bands indicates that they were deposited during higher temperatures than the pink bands, whereas the δ^18^O values show the reverse pattern, with a temperature difference between the bands of 4°C. Mg/Ca ratio is a commonly used palaeotemperature proxy dependent upon the increased uptake of magnesium into skeletal calcite elements at higher temperatures [12]. Whilst the ratio can be used to provide absolute temperatures, this relies on well constrained knowledge of sea water composition at the time and can also depend on other factors such as salinity and pH [23, 24]. Salinity also has an impact on the interpretation of δ^18^O data. The δ^18^O of pristine marine carbonate, if precipitated in isotopic equilibrium, is a function of the formation water δ^18^O (controlled by salinity near the coast and global ice volume over longer time scales [25]) and the temperature at the time of the carbonate formation [13]. If *S. jurassica* lived in normal marine salinity waters then we would expect changes to be a function of seasonal changes in seawater temperature, assuming a constant seawater δ^18^O. However, since we do not know the salinity tolerance of *S. jurassica*, lower δ^18^O in the pink bands could potentially also indicate an influx of freshwater, perhaps due to greater rainfall and river flux. That being said, there is some evidence that, some marine organisms such as forams show a wide range in Mg/Ca ratio at the same depth [12]. It may therefore be the case that our range of Mg/Ca ratios is simply too small to allow us to say anything meaningful about the temperature range of this specimen, given that the data come from a single specimen and depth range is limited.

The clear cyclicity of δ^13^C is more useful in determining seasonal patterns, however its interpretation is also complex. In marine environments the main sources of carbon are CO_2_ and the dissolved inorganic carbon (DIC) ion. Changes in δ^13^C are thought to reflect processes such as changes in salinity (in coastal and estuarine environments), with marine DIC having a higher δ^13^C than freshwater [26], or changes in the rate of photosynthesis [27, 28]. Higher δ^13^C is therefore possibly a result of increased marine salinity, or greater productivity due to seasonal variation. Overall, if it could be assumed that the carbonate has not recrystallized, the white banding suggests cool or more marine conditions from δ^18^O and freshwater or lower photosynthesis from δ^13^C. The pink banding suggests warmer or greater freshwater flux from δ^18^O and more marine or higher photosynthesis from δ^13^C. The increased levels of chlorine in the white bands shown by SRS-XRF (Fig 8) may be taken as an indication that there was increased salinity during the time the white layers were deposited. EPMA (Table 1) (XRF (Table 4) and XPS (Table 4) point analyses are consistent with the pattern shown via SRS-XRF, and indicate that chloride content is approximately a factor of two higher in the white bands when compared to the pink. Given the above, the δ^18^O and Cl levels would indicate more marine conditions in the white band and greater freshwater flux in the pink band, whereas the δ^13^C would suggest higher photosynthesis in the pink bands than the white bands.

Despite such caveats the combination of data from these techniques clearly indicates that there is some seasonal variation in the palaeoenvironment that *S. jurassica* inhabited.

### Palaeoenvironmental implications

Whilst the nature of the environment that *S. jurassica* inhabited has been fairly well described as warm shallow seas, this study has provided another line of evidence that the area was most likely seasonally variable. Current knowledge of the palaeoenvironment comes from geological studies as well as from the presence of fossil organisms typical of such environments, such as gastropods, corals and echinoids [1]. We have been able to demonstrate that the organisms in this area were also subjected to some type of seasonal variation, however the specific nature of the variation will require more work to narrow down. Given that the area at the time was fairly tropical it may seem unusual to have any significant seasonal variation in temperature, so therefore it may be more likely that the variation might be in another factor such as salinity, which would make sense given the significant variation in Cl we see in the bands of *S. jurassica*.

### Mechanism of differential cellular preservation

The cellular structure observations (Fig 1) are consistent with previous reports [1–3] that the visible banding on the sample corresponds to a difference in cellular preservation, with the white bands containing significantly better preserved cells than the pink bands. The current mechanism proposed for this differential preservation [2] predicts that there will be higher magnesium levels in the calcite of the pink bands, causing a loss of cellular detail as it reverts to lower magnesium calcite over time, presumably leading to an equalisation of magnesium levels across the bands. In UOM-232 the magnesium levels are found to be significantly higher in the pink band than the white band vacuole, however the level in the white band cell wall was significantly higher than both (Table 1). As the bands are mineralogically identical (Fig 10), it would not be unreasonable to assume that during diagenesis magnesium levels across the bands, if anything, would have equalized. If the mechanism were accurate we would therefore expect to see some indication of degradation in the white band. Given that the magnesium seems to occur preferentially within the cell walls (Fig 2) it may be that cellular degradation in the pink band by another mechanism led to a significant loss of magnesium from this band over time. Given the high levels of magnesium in the white band coupled with no indication of associated cellular degradation we currently find no evidence in support of the mechanism previously proposed [2].

### Pigment preservation and affinity

The presence of boron within *S. jurassica* could not be verified using XPS. The shift in peak position caused by the presence of phosphate obscures any potential boron peak, even when the sample is scanned at high resolution (Fig 3). This indicates that if there is any boron present it is at levels lower than those of phosphorus, probably at the ppm level.

The TLE of the UOM-232 bulk material was colourless, indicating that the organic pigment was largely retained in the kerogen, which turned brown after calcite removal. Py-GCMS analysis of both bulk and kerogen material after thermochemolysis (Fig 6) revealed the presence of protein moieties (dimethylamino phenol and the amino acid phenylalanine) and tetrapyrrole methane. Infrared analysis also indicates the presence of protein (NH, NH_2_) and carboxylic acid (COOH) groups (Fig 7), though some of these peaks are convolved making identification difficult. Whilst these compounds individually may be found in other pigments and organic compounds, the combination seen here is consistent with the presence of the red pigment phycoerythrin.

Phycoerythrin is a pigment that occurs attached to protein and is therefore known as a phycobiliprotein. It is found in cyanobacteria and certain algae [29] and is a photosynthetically active light harvesting complex composed of a backbone of open chain tetrapyrroles bonded to various functional groups including carboxylic acids [30, 31]. Phenylalanine is a component amino acid of the phycoerythrin-protein complex, however it is not the most abundant [32]. Therefore, whilst detection of pheylalanine is consistent with the presence of phycoerythin, more detailed proteomic analysis should be used to determine the details of the amino acid inventory of *S. jurassica* and to see whether it varies across the different bands.

Py-GCMS analysis reveals no biomarkers indicative of bacteria (hopanes), algae (steranes) [33] or sponges (24-isopropylcholestane [34]), however the apparent presence of the pigment phycoerythrin is taxonomically indicative, and given that there is no evidence in this study or any previous one for the presence of bacteria the classification of *S. jurassica* as a calcareous alga is most likely to be correct.

## Conclusions

The presence of density banding demonstrates that the visual bands seen in *S. jurassica* are most probably seasonal growth bands. The cyclical pattern of δ^13^C also indicates a degree of seasonality though its precise interpretation is at this stage unclear. The same is true of the temperature during the deposition of the different bands, with Mg/Ca ratios indicating that the white bands were deposited during higher temperatures than the pink bands, and the δ^18^O potentially suggesting the opposite. Higher temperature in the white bands is supported by the presence of higher levels of chlorine in the white bands, possibly originating from increased evaporation due to the higher temperatures, however this alone is not enough to suggest temperature difference. The identification of Pyrrole tetramethyl, protein moieties and carboxylic acid groups by Py-GCMS and FTIR are together suggestive of the presence of the red algal pigment phycoerythrin. This would support the identification of *S. jurassica* as an alga as phycoerythrin occurs only in cyanobacteria and algae; there is no biomarker evidence of input from bacteria or sponges. Boron, from the previously identified borolithochrome pigment, could not be determined as it likely occurs below the ppm level. The mechanism of differential cellular preservation by differences in magnesium uptake is not supported.

Further work should be done to determine whether there are patterns in pH in the bands, as it has been shown in modern calcareous Coralline algae that the combination of high UV radiation levels and atmospheric CO_2_ led to the inhibition of growth, calcification and caused pigment degradation in certain species [35]. The determination of Sr/Ca molar ratios could also help to determine the specific temperatures and salinities present in each band. In addition, proteomics analysis would help to determine the amino acids present in *S. jurassica* and see whether they covary with the bands, and artificial maturation of extant red calcareous algae would help to understand the degradation pathways of the phycoerythrin pigment.

The potential presence of a taxonomically specific pigment which aids the classification of *S. jurassica*, indicates that such a method could be used for classifying other potentially problematic fossils. Though it would only be applicable if sufficient quantities of original pigment had been preserved. The use of X-ray radiography to study seasonality however, has the potential to be more widely useful. The shift in density does not have to correlate to a pigment change, and may well indeed be completely invisible under natural light. Other calcareous algae, indeed any fossil that lays down an external calcareous skeleton could be analysed in the same way, as corals have been in the past.

## Supporting Information

S1 Fig EPMA maps of the pink and white bands of *S. jurassica*. White represents high abundances and black represents low abundances.
(TIFF)

S2 Fig Synchrotron rapid scanning X-ray fluorescence maps of the ‘lighter’ elements in UOM-232 with a photographic reference of the area scanned. White represents high abundances and black represents low abundances.
(TIFF)

S3 Fig Synchrotron rapid scanning X-ray fluorescence maps of the ‘heavier’ elements in UOM-232 with a photographic reference of the area scanned. White represents high abundances and black represents low abundances.
(TIFF)
