# Supplementary figures and images for: Geochemical Evidence of the Seasonality, Affinity and Pigmenation of Solenopora jurassica
Source: PLoS One. 2015 Sep 14;10(9):e0138305. doi: 10.1371/journal.pone.0138305 (PMC4569467; doi:10.1371/journal.pone.0138305)

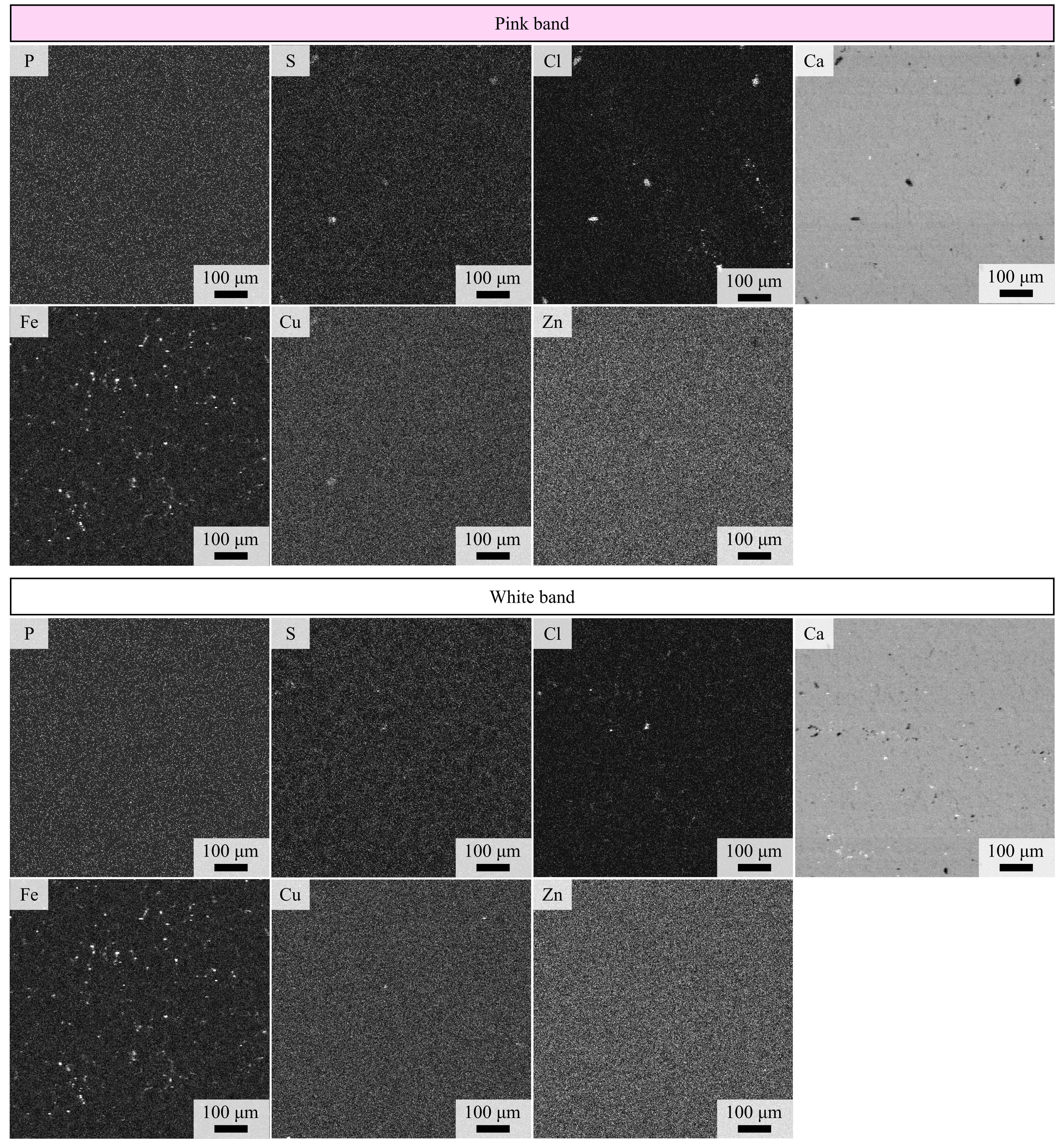

Supplement: S1 Fig — White represents high abundances and black represents low abundances. (TIFF) [file pone.0138305.s001.tiff]

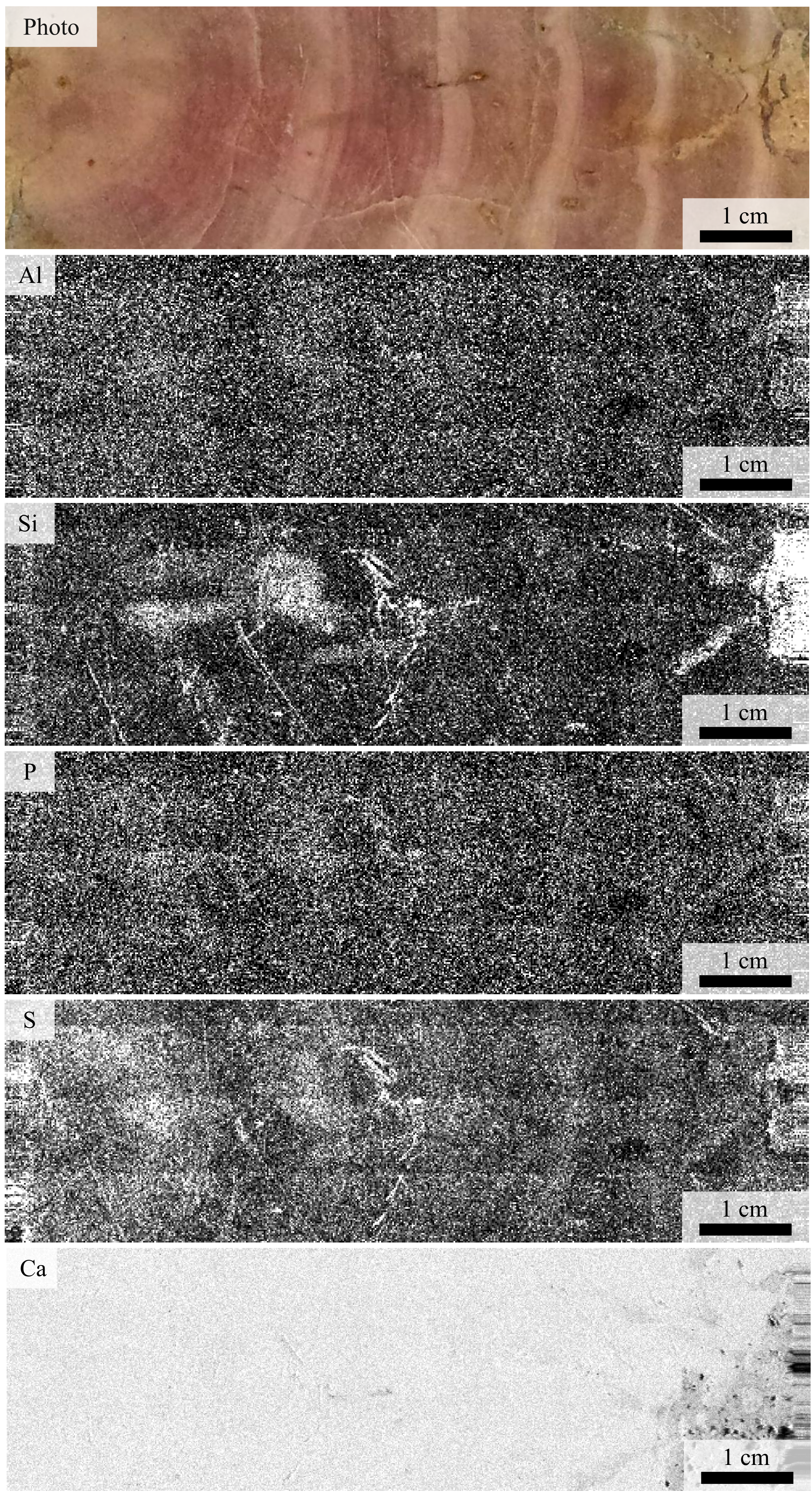

Supplement: S2 Fig — White represents high abundances and black represents low abundances. (TIFF) [file pone.0138305.s002.tiff]

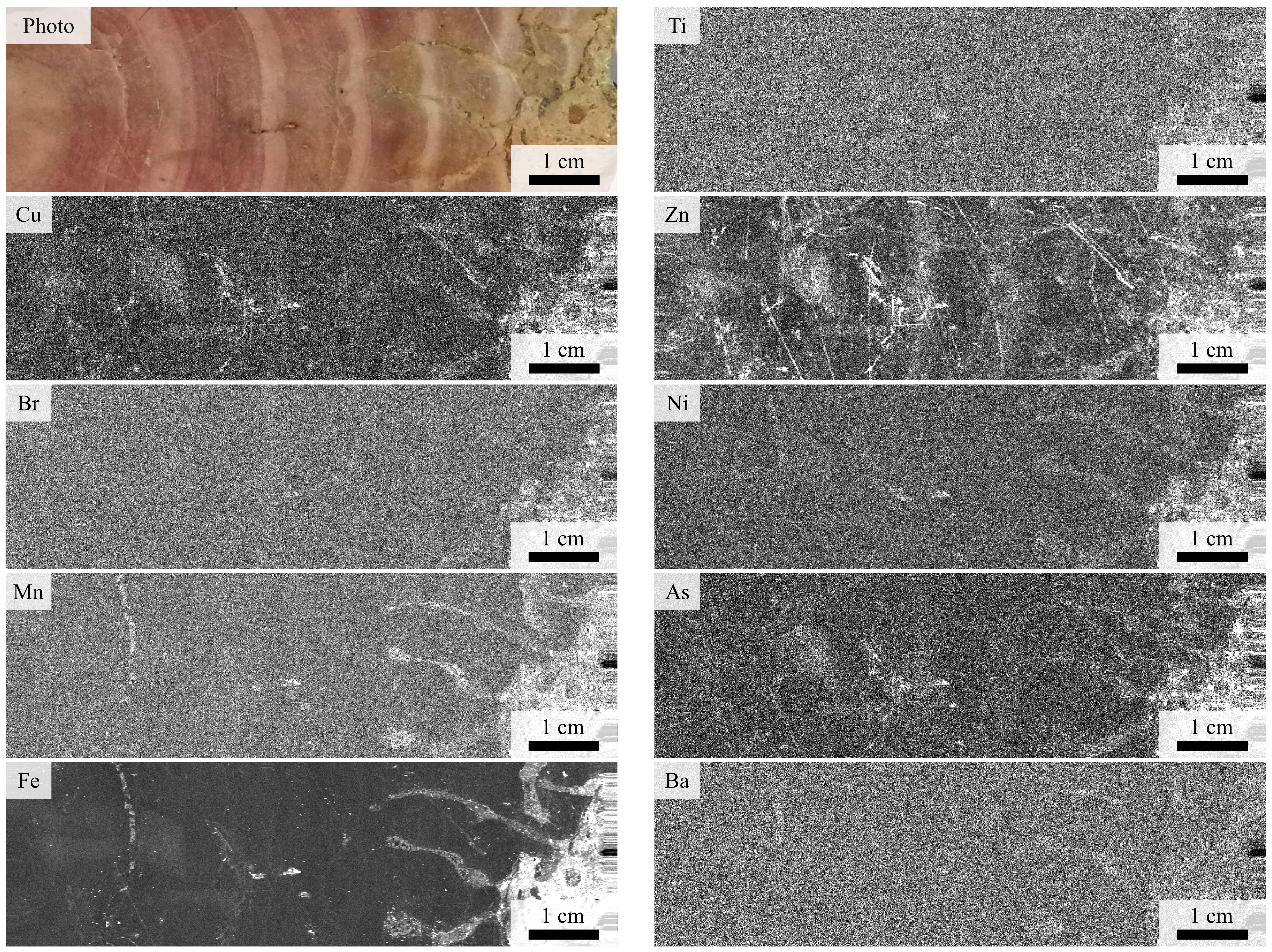

Supplement: S3 Fig — White represents high abundances and black represents low abundances. (TIFF) [file pone.0138305.s003.tiff]
